# Exercise to transform tumours from cold to hot and improve immunotherapy responsiveness

**DOI:** 10.3389/fimmu.2023.1335256

**Published:** 2023-12-12

**Authors:** Brindley Hapuarachi, Sarah Danson, Jon Wadsley, Munitta Muthana

**Affiliations:** ^1^ University Sheffield, Division of Clinical Medicine, Sheffield, United Kingdom; ^2^ Weston Park Cancer Centre, Sheffield Teaching Hospitals National Health Service (NHS) Foundation Trust, Sheffield, United Kingdom

**Keywords:** exercise, tumour, immunity, microenvironment, immunotherapy, hot, cold

## Abstract

Exercise provides significant health benefits to patients diagnosed with cancer including improved survival outcomes, quality of life and reduced cancer recurrence. Across multiple murine cancer models, aerobic exercise and resistance training has exhibited anti-tumour properties illustrated by inhibited tumour growth, reduced metastatic potential and modulation of the tumour microenvironment to allow the recognition and destruction of cancer cells. Clinical studies have demonstrated the rapid mobilisation and circulatory release of mature lymphoid populations, myokines and cytokines that occurs with exercise along with tumour vasculature normalisation. Tumour microenvironments enriched with immune cells with anti-cancer potential, such as CD8+ T cells, are termed ‘hot’, whilst those favouring an immunosuppressive environment and lacking in effector immune cells are classed as ‘cold’. Pre-clinical evidence suggests exercise training has the potential to reprogramme cold tumours to become hot, although this requires validation in clinical studies. This hot environment could potentiate immunotherapy responsiveness, improving survival outcomes of patients undergoing cancer immunotherapy and allow those with typically cold tumours to benefit from immunotherapy. This review discusses the complex interactions between exercise and cancer, including exercise-induced alterations within the tumour microenvironment and systemic immunity. The potential role exercise may play in improving cancer immunotherapy responsiveness is explored. This review also highlights the need for translational studies exploring the role of exercise in patients with cancer with the potential to widen the spectrum of tumours that derive significant benefit from immunotherapy.

## 1 Introduction

### 1.1 The immune climate of the tumour microenvironment: cold vs hot

Immunotherapies, such as immune checkpoint inhibitors (ICIs), have significantly extended survival outcomes across multiple different cancers. Immune system evasion is a key tactic utilised by cancer cells to allow their growth, migration and invasion. ICIs facilitate cancer cell recognition and immune cell destruction by inhibiting pathways they use to evade the immune system. Although ICIs have demonstrated significant benefits in cancer care, they are limited by a spectrum of potentially serious side-effects, including fatigue and immune-mediated toxicities such as hepatitis, colitis and pneumonitis (1). Fewer than 40% of patients derive significant benefit from ICIs which highlights the need to better understand the mechanisms for resistance to immunotherapy and how to overcome this to improve responsiveness (1, 2). ICI response correlates with the degree of immune cell infiltration within the tumour microenvironment (TME), which varies significantly between tumours on the cold to hot spectrum.

When a TME lacks effector immune cells such as CD8+ T cells and Natural Killer (NK) cells, as in the cases of colorectal and pancreatic cancer, this is considered a ‘cold tumour’ (3). Myeloid derived suppressor cells (MDSCs) and tumour associated macrophages (TAMs) can be found in abundance within cold tumours creating an immunosuppressive environment and consequently, tumours are able to propagate without significant resistance from the host immune system (3–5). This less favourable immune profile correlates to a limited, if any, response to ICIs (4, 5).

In comparison, ‘hot’ tumours, such as melanoma, are highly immunogenic with a rich CD8+ T cell population within their TME (5). Therefore, hot tumours hold the potential for immune-mediated recognition and destruction of tumour cells, which cold tumours inherently lack. The abundance and potential anti-cancer activity of these immune components can vary between different hot tumours, partly explaining the varying responses to ICIs amongst hot tumours. Dual ICIs with nivolumab and ipilumumab have some success in the treatment of advanced mesothelioma, which could be classed as a hot tumour due to the presence of immunogenic cells within the tumour microenvironment, although only 40% respond with a median overall survival of 18.1 months (6). This is a relatively modest benefit when compared to ICI response to a classically hot tumour such as melanoma, where the overall survival exceeds 60 months with dual ICIs (7). The TME in mesothelioma is complex with NK cell, CD4+ and CD8+ T cell infiltration supporting its classification as a hot tumour, however functional aberrations prevent their full utility (8). This coupled with high concentrations of pro-tumour TAMs suppress the degree of responsiveness seen with ICIs (8). This implies that tumours lie on a spectrum from cold to hot with some tumours exhibiting both cold and hot characteristics.

There is a growing body of evidence that exercise training can modulate local tumour and systemic immunity in patients with cancer, skewing the immune profile to favour anti-tumour activity. Exercise training could be a simple, safe, cost-effective method to help shift tumours towards the hot end of the spectrum and to support ICIs’ mechanism of action. This would lead to improved responses and survival outcomes in patients with hot tumours undergoing immunotherapy, as well as expanding the range of cancers that can obtain benefit from immunotherapy, such as pancreatic cancer.

Furthermore, exercise has known health benefits including enhancing quality of life and ameliorating adverse effects associated with cancer and its treatment (2, 3). The evidence for the effect exercise exerts on different tumour types within the cold-hot spectrum via different mechanisms is described in detail below.

## 2 Exercise and cancer

The definition of physical activity is “*any bodily movement produced by skeletal muscles or that requires contraction of your muscles and energy expenditure*” (9). In order to be classed as exercise, activity would need to consist of organised, repeated movements which when done regularly, can have a positive impact on cardiovascular and respiratory function, physical fitness and general overall health (9).

Exercise has been shown to have health benefits in the general population, both physically by improving cardiovascular, respiratory and musculoskeletal functioning and mentally (9). The United Kingdom National Health Service (NHS) and the American College of Sports Medicine (ACSM) guidelines recommend regular aerobic exercise (150-300 minutes of moderate intensity or 75-150 minutes of high intensity per week) and resistance training twice weekly (10, 11). These recommendations are provided for the general population, however there is currently no tailored exercise advice to patients with advanced cancer. Given the wide range of functional abilities and background health conditions, a ‘one size fits all’ exercise regime would not be appropriate.

Evidence shows that the added benefits in patients with cancer include improving overall survival, reducing cancer-related and treatment induced fatigue along with reducing cancer recurrence (4, 12). It would be prudent to define the exercise event to provide a reproducible, standardised intervention in cancer care (4). Acute exercise includes independent exercise activities compared to regularly repeated exercise lasting months with exercise training or more than a year with chronic exercise (4). Studies mainly correlate exercise training at moderate intensity aerobic exercise with the physical and mental health benefits seen in cancer patients (13, 14). Furthermore, the FITT criteria (Frequency, Intensity, Time and Type) would provide information on dosing required to exhibit intended benefits and does require further research in order to cater to the advanced cancer population (4).

### 2.1 Cold tumours

Colorectal cancer is classically a cold tumour when it possesses microsatellite stability rendering ICIs ineffective (15). There is strong evidence that exercise has a protective role against colorectal cancer with an estimated relative risk reduction in colorectal cancer development of 12 to 28% (16). One of the proposed mechanisms behind this is the exercise-induced reduction in adipose tissue and the metabolic benefits including optimising insulin sensitivity, although studies suggest other pathways contribute to the reduced cancer risk (16). Although the indirect mechanisms described above are well documented, the direct anti-cancer processes induced by exercise require further exploration.

Zylstra et al. reviewed the effect of incorporating combined moderate intensity supervised and home-based aerobic exercise activities into the pre-operative treatment pathway, consisting of multi-agent neoadjuvant chemotherapy, for potentially resectable oesophageal cancer, which is a cold tumour (13). This single centre controlled study showed that the exercise cohort had a significantly enhanced tumour response to treatment compared to controls (13). This was demonstrated by a higher rate of Mandard Tumour Regression Grades (MTRG) of 1-2 (13). MTRG is a scoring system from 1-5, where a score of 1 indicates no evidence of active cancer cells (complete response) and a score of 5 suggests no evidence of cancer regression (no response) within the pathology specimen. There are multiple limitations to this study including non-randomisation due to geographical issues in delivering supervised exercise activities, small sample size and the modification in clinical guidelines during the study with regards to optimum chemotherapy regimen (13). Given the relatively poor prognosis of oesophageal cancer and limited treatment options, a robust randomised controlled trial is required to support exercise as an effective anti-cancer intervention and determine the effect on more relevant clinical endpoints such as overall survival.

### 2.2 Hot tumours

Exercise significantly inhibits tumour development and growth in preclinical models of hot tumours including transplanted Lewis lung cancer, diethylnitrosamine induced liver cancer and melanoma (GrM1) mouse models (17). Pedersen et al. (12) demonstrated anti-cancer benefits in female mice that performed exercise training over four weeks. This involved wheel running at an average of 4.1km a day for each mouse, before both subcutaneous implantation and intravenous administration of B16F10 melanoma cells (12). A statistically significant 61% reduction in tumour size and lower incidence of metastatic lung disease in the exercised mice were seen (12). This demonstrates exercise plays a dual role in primary tumour inhibition and prevention of metastases.

Only female mice were inoculated with this model introducing an inherent gender bias. Although the positive effect of wheel running on cancer regression was also demonstrated in five different murine models including diethylnitrosamine induced liver cancer in male Naval Medical Research Institute (NMRI) mice (12). To further explore the mechanism behind the inhibited tumour growth in the B16F10 melanoma model, microarray analysis confirmed increase in immune cell activity with enhanced gene activation, cytokine expression and immune cells both of pro-inflammatory and anti-inflammatory nature (12). Chronic inflammation correlating with increased expression of pro-inflammatory immune components can be associated with the carcinogenic process. Microarray analysis specifically noted increased interleukin (IL)-1α and inducible nitric oxide synthase (iNOS levels) (12), both involved in signalling pathways that promote tumour growth and a raised IL-1α level was shown to be a poor prognostic factor in gastric cancer and squamous cell carcinoma affecting the head and neck (18, 19). Despite the upregulation of these pathways, an overall reduction in tumour growth and incidence suggests exercise induces a series of complex immune processes, which favour anti-tumour activity.

## 3 Exercise and immunity

Evidence is increasing in support of the role of exercise in modulating the immune system and TME through multiple mechanisms in cancer patients (4). ‘Exercise-induced leucocytosis’ refers to the immediate increase in circulatory leukocytes after a single exercise activity (20). Murine models have demonstrated that exercise up-regulates immune pathways in tumours including natural killer (NK) cells, B cells, T cells and dendritic cells (17). NK cell circulatory release appears to be the most sensitive and have an immediate response to acute exercise driven by catecholamine release (17). NKG2D and NKp46 are NK cell activating receptors that are upregulated within the TME with exercise (21). Within the hour after exercise cessation, T cells continue to produce cytokines and *in vitro* studies have shown NK cells are more efficiently cytotoxic against myeloma and lymphoma cell lines (4, 22). Improved outcomes can be seen across many different cancers with an NK cell rich TME likely due to their cytotoxic function (21). Pederson et al. were able to show in the B16F10 melanoma murine model, lower tumour burden was associated with raised NK cell tumour infiltration (12). They were able to demonstrate tumour specificity of NK cell concentration as opposed to lymphoid organ NK cell accumulation seen in exercised control mice (12). This highlights that exercise-induced immune responses can be directed to local tumour immunity rather than a non-specific generalised mechanism and help to create a hot TME.

Despite this well evidenced exercise driven leucocytosis, the converse depletion of NK cells and CD8 T cells below baseline levels is observed after three hours of exercise completion (23). However, this lymphopaenia may reflect their redistribution from circulation to peripheral tissue supporting a continued enhancement of immune function (23).

A high abundance of NK cells within the systemic circulation and intratumourally has been correlated with a favourable prognosis and specifically, a reduced metastatic potential in a range of hot to cold tumours including renal cell carcinoma (hot) and gastric cancer (cold) (24). Davis et al. correlated improved survival outcomes in patients with pancreatic cancer with higher circulatory NK cell count (25). Metastatic disease in all solid malignancies is reliant on the ability of cancer cells to evade regulated immune-mediated destruction during separation from the primary tumour, manipulation and restructuring of the extracellular matrix and movement through the circulatory system (24). CD8+ T cells, effector cells of adaptive immunity, rely on presentation of tumour antigen on MHC class I molecules, thus NK cells are able to act synergistically to eliminate cancer cells that lack MHC class I expression (26). Utilising this exercise-induced burst of NK cells may be of particular benefit to in reducing the metastatic potential of tumours.

**Figure 1:**
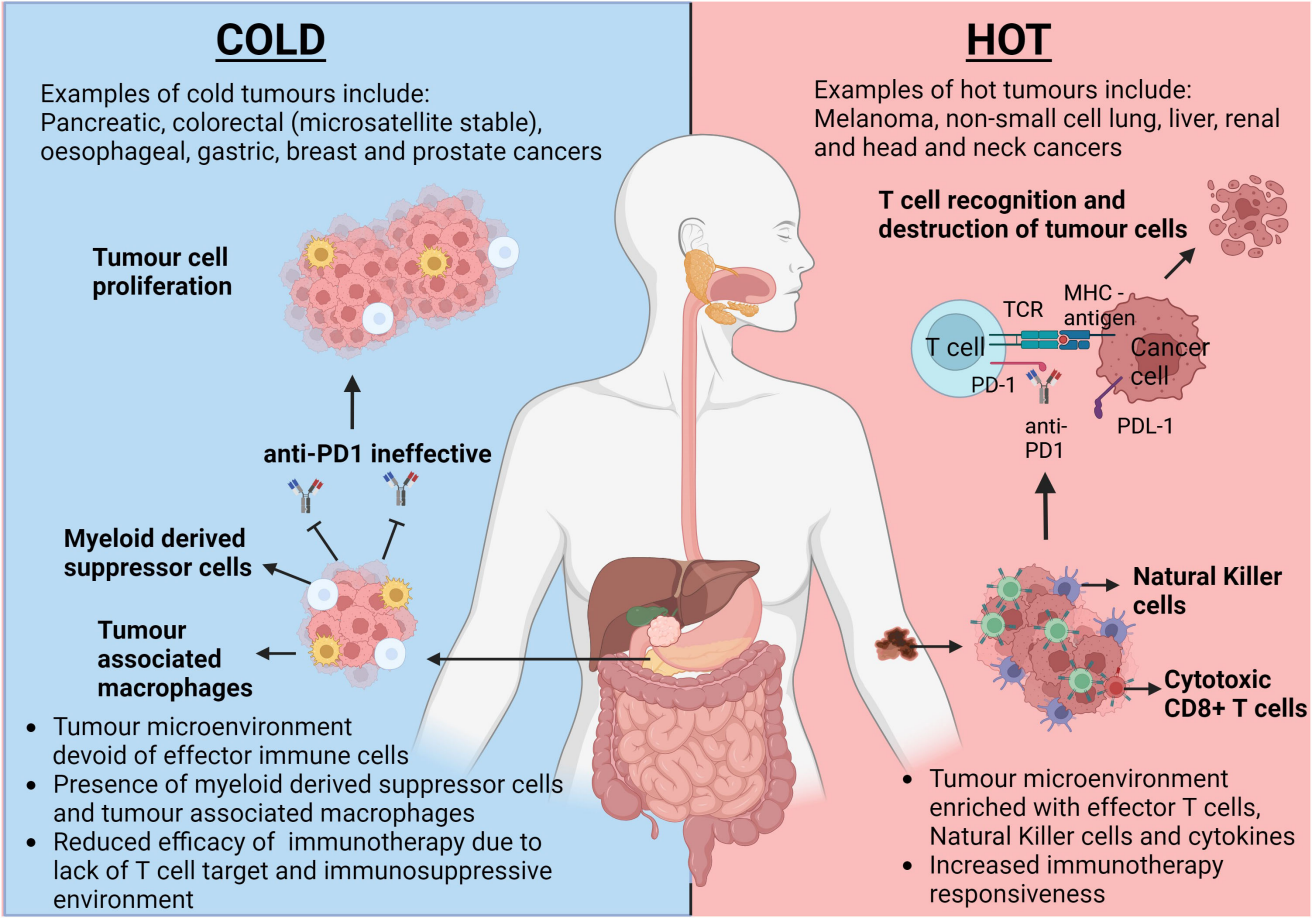
The opposing responses to immunotherapy dependant on the tumour microenvironment climate. This figure highlights the differences in tumour microenvironments (TMEs) between a classically hot tumour such as melanoma and a classically cold tumour such as pancreatic cancer. Intratumoral T cells in hot tumours provide a target for immunotherapies, such as anti-PD1, to allow inhibition of PD-1/PDL-1 interaction and allow subsequent cancer cell lysis. Due to the inherent lack of T cells within cold tumours, tumour cell proliferation remains uninhibited. TCR, T cell Receptor; MHC, Major Histocompatibility Complex; PD-1, Programmed Cell Death Protein 1; PDL-1, Programmed Cell Death Ligand 1. Created with BioRender.com

Importantly, many studies showing correlation between exercise and increasing immune cell tumour infiltration in murine models have been undertaken in hot tumours with TMEs rich in tumour inhibiting T cells and cytokines such as interferon gamma (IFN-γ) (5) and therefore, inherently immunogenic. Multiple diverse mechanisms can result in systemic immune dysfunction and cold tumours such as pancreatic and prostate cancer, have been shown to have low immune cell populations and reduced immune activity within their TMEs as demonstrated in **Figure 1** (4, 5).

Cancer cells are able to survive and proliferate by hiding from the immune system and preventing destruction by a number of different mechanisms (21). One of these mechanisms seen in pancreatic ductal adenocarcinoma (PDA), the most common subtype of pancreatic cancer, is reconfiguring immune cell production with consequent proliferation of myeloid cells, with a preference towards an immature subset and a reduction in lymphoid cells that exhibit anti-cancer activity, subsequently promoting an immunosuppressive environment (27). The paucity of anti-tumour immune cell concentrations in PDA and consequent lack of responsiveness to immunotherapies, highlight the specific need to optimise local tumour and systemic immunity in cold tumours.

Schwappacher et al. identified statistically significant changes in PANC-1 (primary human pancreatic cancer cell) viability following incubation with serum taken from pancreatic cancer patients at baseline, 6 and 12 weeks into a whole body electromyostimulation (WB-EMS) programme; a method used to simulate the effect of resistance training (28). Supporting this, Kurz et al. demonstrated exercise induced benefits within different PDA mouse models showing tumour mass was decreased by 20-30% in exercised mice with their methodology accounting for potential confounding factors of reduced body and muscle mass (27). The reasoning behind this was explored using single cell RNA sequencing, demonstrating that exercise caused the shift towards mature lymphoid populations, such as cytotoxic CD8 T cells whilst reducing MDSCs within the pancreatic TME (27), thereby reducing the immune evading ability of the tumour. Exercise reduced CXCR2 expression, which is a myeloid cell receptor involved in signalling pathways that enhance MDSC populations (27).

Ex vivo studies demonstrated higher Ki-67 levels, indicating proliferative activity, in CD8 T cells when cultured with MDSC isolated from tumours from exercised mice compared to controls indicating increased T cell activity and a reduction in the MDSC immunosuppressive effect (27). To the best of our knowledge, this currently is the only mouse study demonstrating the direct anti-cancer activity of exercise on PDA and its ability to turn this cold tumour hot. Given the poor prognosis, survival outcomes and high morbidity and mortality, this warrants further evaluation including in human studies.

A favourable immune environment has also been demonstrated in human studies with significantly raised CD3+ and CD8+ T cells and lowered Tumour Necrosis Factor alpha (TNFα) levels seen in the exercise cohort of oesophageal cancer patients in the study by Zylstra et al. (13). These studies have demonstrated the promising abilities of exercise to allow effector T cell infiltration and MDSC depletion within cold TMEs creating hot tumours.

## 4 Exercise and myokines

Myokines, including IL-6, IL-7 and I-15, released on muscle contraction play a role in immune system mediation along with many other physiological processes and therefore play a critical role in exerting the systemic effects of exercise (4, 29). As discussed before, many components of the immune system can exhibit opposing mechanisms of action. IL-6 is involved in both pro-inflammatory and anti-inflammatory pathways, and it is the mechanism of release along with local and systemic conditions and duration of exposure which skew the direction of immune activity with exercise-induced short-term rises in IL-6 demonstrating more of an anti-inflammatory effect (4). One *in vitro* study using LoVo cell line (colorectal cancer cell line KRAS mutant TP53 wildtype) incubated with human serum taken pre and post exercise, showed a 4.2% reduction in cell proliferation following exercise and demonstrated a 24.6% rise in serum IL-6 post exercise (16). The study demonstrated, using recombinant IL- 6 on LoVo cells, that increasing IL-6 led to a proportionate decrease in LoVo cell proliferation and γ-H2AX expression which relates to DNA damage (16). This study supports exercise-induced IL-6 as having potential anti-tumour properties although the complex dynamics and interactions of exercise-induced IL-6 *in vivo* would need to be examined and compared to the pro-inflammatory carcinogenic and immunosuppressive actions associated with long-term exposure to IL-6, which has been documented in murine models as enhancing CCR5 expression and subsequent promotion of MDSC activity (30).

IL-7 and IL-15 play an important role in modulating T cell levels and activity and are released with muscle contraction during exercise (4). IL-7 is involved in early signalling pathways stimulating naïve T cell proliferation and along with IL-15, promotes effective memory T cell concentrations after exposure to antigen (4). Kurz et al. (27) demonstrated the key anti-cancer role exercise-induced IL-15 plays by blocking IL-15 downstream signalling in PDA models *in vivo* and negating the beneficial effects. Therefore, the release of IL-7 and IL-15 during exercise may help shape the immune landscape and turn cold tumours hot.

There have been studies identifying different, novel myokines which exhibit anti-tumour properties, such as the secreted protein acidic and rich in cysteine (SPARC) myokine, oncostatin M (OSM) and irisin (31–33). SPARC levels rise instantaneously with acute exercise with a gradual decline over six hours following exercise cessation (31). Kim et al. demonstrated a significant rise in myokines oncostatin M and SPARC in patients with metastatic castrate resistant prostate cancer (mCRPC) who underwent a 6 month supervised exercise regimen combining resistance training with aerobic exercise (32). Colon-26 cancer cell proliferation was suppressed when mouse recombinant SPARC was added (31) and there was reduced cell growth *in vitro* when DU145 prostate cancer cells were cultured with their myokine enriched serum compared to the control arm (32). However, the suppression plateaued at SPARC concentrations higher than 2 µg/ml and it is therefore difficult to conclude that raised SPARC levels above baseline in humans would have a similar effect.

Limitations include small sample size and hence an inadequately powered study and the lack of specificity correlating the rise in myokines and their subsequent signalling pathways to tumour inhibition, highlighting the need for further review of the potential cytotoxic mechanisms in prostate cancer with exercise induced myokines (32).

**Figure 2:**
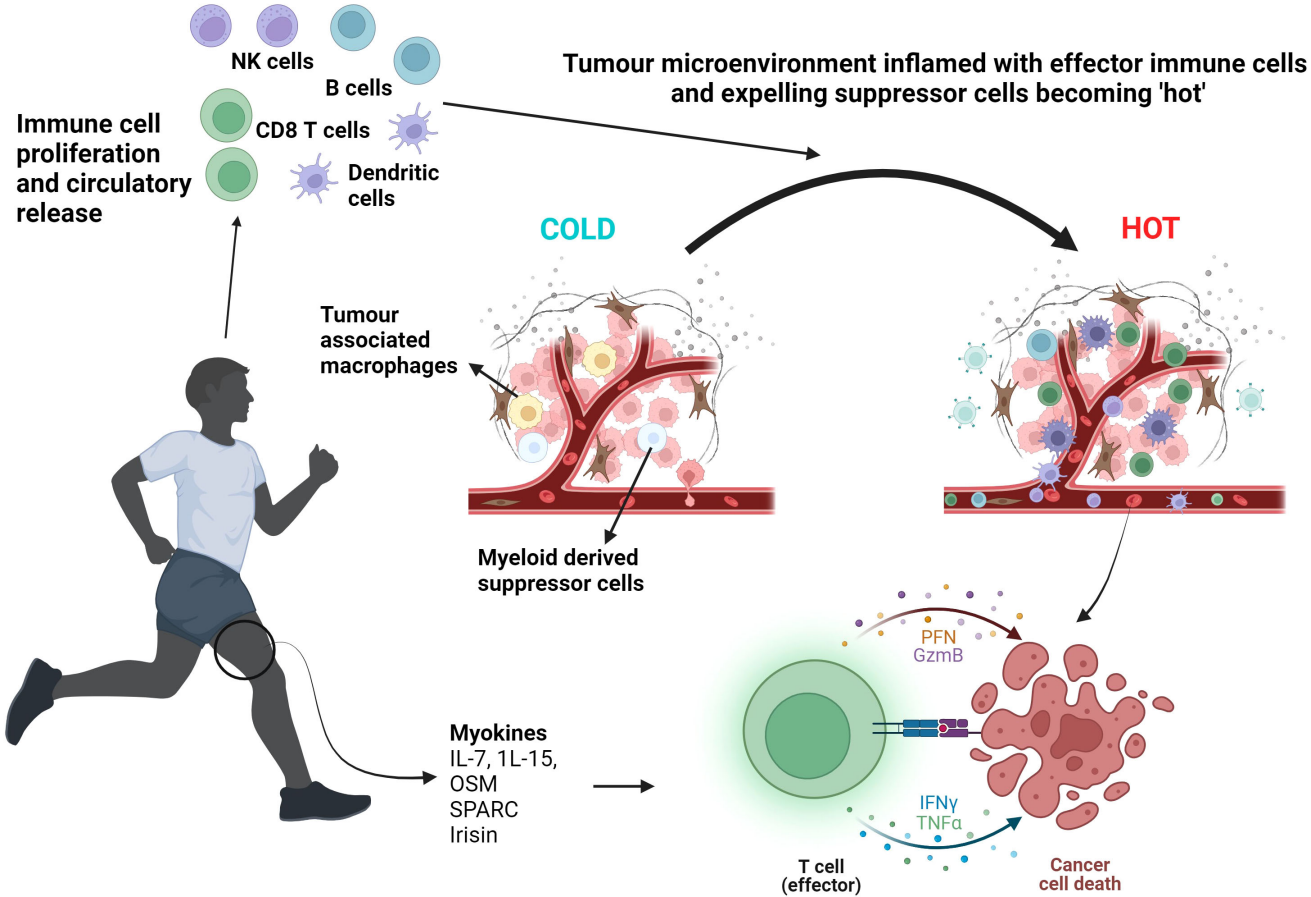
Exercise induced myokine and immune cell proliferation and release leading to T cell activation and cancer cell death. Aerobic exercise and resistance training mobilise myokines such as IL-7. IL-15, OSM, SPARC and irisin alongside inducing a circulatory leucocytosis, including NK cells and T cells. The TME becomes ‘inflamed’ with effector immune cells. Effector T cell function is enhanced resulting in tumour lysis. NK cells, Natural Killer cells; OSM, Oncostatin M; SPARC, Secreted Protein Acidic and Rich in Cysteine; PFN, Perforin; GzmB, Granzyme B; IFNγ, Interferon gamma; TNFα, Tumour Necrosis Factor alpha. Created with BioRender.com

Irisin is another myokine which exhibits significant metabolic effects leading to increased energy expenditure with an immediate increase in circulatory levels demonstrated following both aerobic exercise and resistance training (33, 34). Gannon et al. showed in an aggressive epithelial breast cancer cell line, a significantly reduced cell number and viability and increased apoptotic signalling with enhanced caspase-3/7 activity with human recombinant non-modified irisin (33). Liu et al. demonstrated irisin’s inhibitory effects on cancer cell growth in pancreatic cancer cell lines (35). This provides convincing *in vitro* evidence behind irisin’s anti-cancer potential although *in vivo* studies would further strengthen the correlation between exercise-induced irisin and tumour inhibition. Schwappacher et al. provide evidence supporting anti-migratory properties of WB-EMS against pancreatic cancer cells implicating myokines, such as IL-10 and CCL4, as promoters of caspases3/7 apoptotic signalling (28). By inhibiting cancer cell migration, required for metastatic disease formation, this further supports the role resistance training-induced myokines play on reducing the metastatic potential of tumours. *In vivo* studies would also allow exploration of how exercise-induced myokines modulate cold TME and prevent metastasis formation. **Figure 2** summarises the effect exercise has on the proliferation and circulatory release of immune cells and myokines leading to the reprogramming of cold tumours to become hot and eventual cancer cell death.

## 5 Exercise and the tumour vasculature

Due to the dysregulated and exponential proliferation of cancer cells, tumour angiogenesis results in an inefficient and flawed vessel structure perpetuating an oxygen-depleted environment and tumour expansion (36). Vascular endothelial growth factor-α (VEGF-α) is associated with angiogenesis and has been shown to be significantly raised in the immunotherapy groups with the combined exercise and nivolumab model in Martin-Ruiz’s study having the highest VEGF-α tumoural levels as well as the highest necrotic index (37). However, there is conflicting evidence with regards to VEGF-α expression and exercise with both an up-regulation and down-regulation reported in the literature (37).

Within the TME, hypoxia fuels a signalling pathway resulting in raised hypoxia-inducible factor-1 (HIF-1) (38). Jones et al. noted a positive correlation between exercise and HIF-1α leading to enhanced VEGF expression within the TME along with ANGPT2, a gene marker of angiogenesis (38). In this study, C57BL/6 mice orthotopically implanted with transgenic adenocarcinoma of mouse prostate (TRAMP) C-1 cells undertook aerobic exercise using a voluntary wheel running method in a study exploring the association between exercise and tumour hypoxia and vessel structure (38).

**Figure 3:**
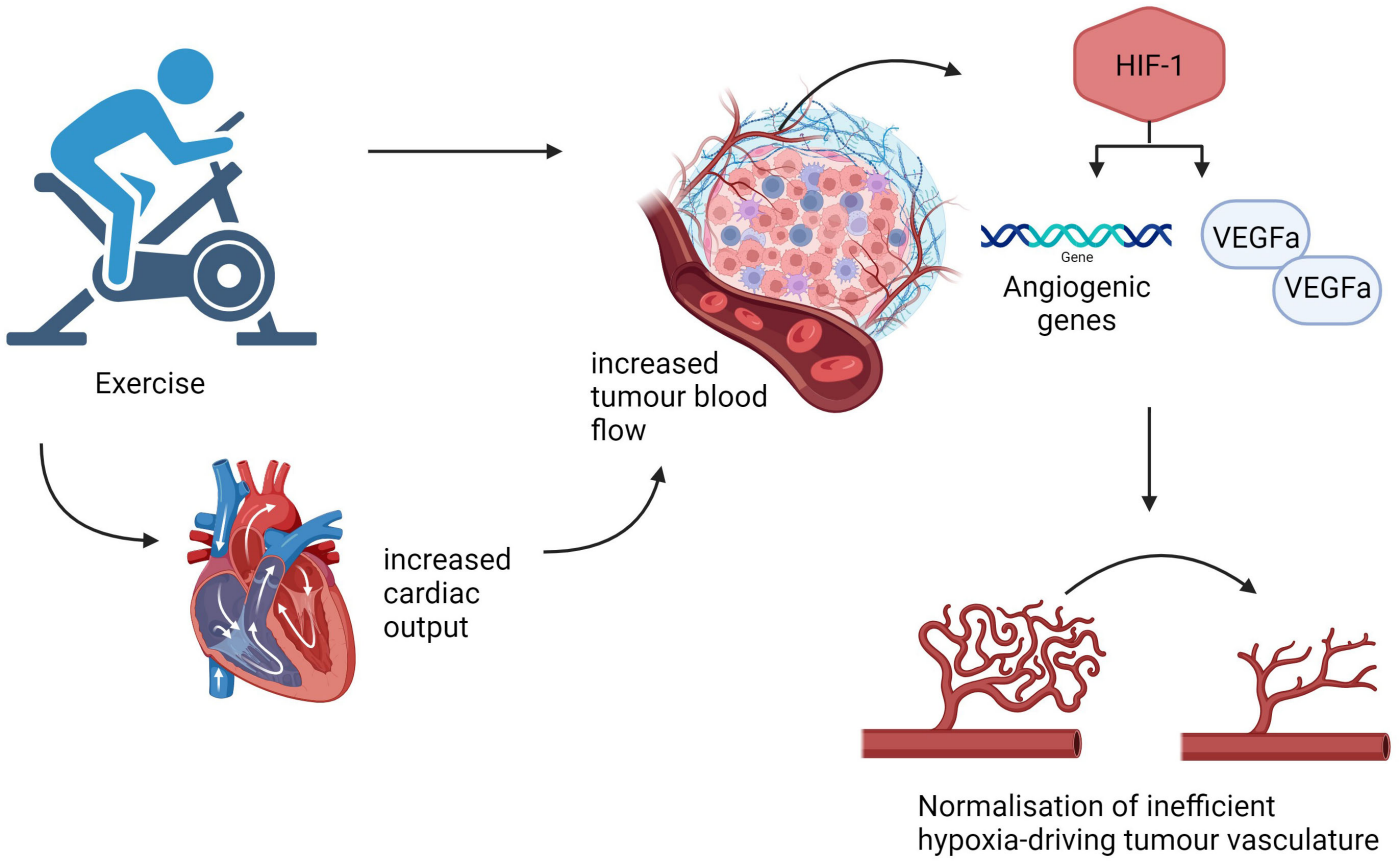
Exercise induced normalisation of tumour vasculature. An outline of some pathways that aerobic exercise may utilise to normalise tumour vessels, increase tumour blood flow and reduce hypoxia. Created with BioRender.com

They demonstrated using dynamic magnetic resonance (MR) scans, a significant increase in blood flow through tumours in exercised mice indicating vasculature normalisation (36, 38). The authors hypothesise that exercise training may be linked to an alteration in the mechanism of action of HIF-1 combined with cytokine release to support tumour blood flow, illustrated in **Figure 3** (38). This suggests tumour vasculature normalisation occurs secondary to the complex, synergistic interactions between exercise and the TME rather than a single pathway. This further supports the theory that exercise leads to enhanced VEGF-α expression and so optimising intratumoural drug delivery (37).

Bedoya et al. demonstrated that a home-based combined aerobic exercise and resistance training regimen prior to surgery for PDA exhibited a favourable change in the tumour vasculature with increased microvessel density and patency (14). Although exercise was performed concurrently with either chemotherapy, combination of chemotherapy and radiotherapy or during the recovery period between pre-operative treatment and surgery, the effect of exercise on treatment responses and clinical outcomes was not explored, subsequently missing a potential translational endpoint (14, 39).

Exercise leads to increased cardiac output and a redistribution of blood flow away from constricted vessels within the splanchnic organs allowing supply to the contracting skeletal muscles (36). Betof et al. demonstrated, using the 4T1 breast cancer murine model, a significant reduction in EF5 (marker of hypoxia) within the tumours of exercised mice compared to sedentary mice (40). One theory behind the proposed benefit of exercise on tumour hypoxia includes the inherent nature of tumour vasculature to ignore vasoconstrictive instructions and consequently take advantage of the raised cardiac output and redirected blood flow (36).

Gomes-Santos et al. identified a simultaneous reduction in tumour hypoxia and an increased CD8+ T cell intratumoural influx with exercise training in mice implanted with breast cancer (41). Furthermore, Hatfield et al. were able to show that increasing the proportion of inspired oxygen in mice reduced tumour hypoxia and allowed a comparable increase in breast tumour infiltrating CD8+ T cells (42). These studies suggest that reduced tumour hypoxia could be a contributing factor to this CD8+ T cell tumour infiltration, providing a favourable hot TME for their stimulation and enhanced function (36).

Therefore, exercise-induced tumour vasculature normalisation of cold tumours could lead to improved oxygenation and CD8+ T cell infiltration indicating a different mechanism of reprogramming cold tumours to become hot. By modulating PD-L1 expression and CD8+ T cell infiltration, exercise-induced tumour oxygenation could potentially improve the efficacy of ICIs working within the PD-1/PD-L1 pathway (36).

## 6 Exercise and immunotherapy

Pre-clinical studies suggest exercise can modulate systemic and local tumour immunity by complex pathways upregulating immune components with anti-tumour activity, turning cold tumours hot and therefore, could play an adjunctive role to immunotherapy in patients with cancer.

Immunotherapy has revolutionised treatments across many different cancers and has shown to be particularly effective in hot tumours (5). ICIs, including anti- Programmed Cell Death Protein 1 (anti-PD1) and anti- cytotoxic T lymphocyte antigen 4 (anti-CTLA-4), are commonly used as standard of care in both curative and palliative settings. However, ICIs are not effective across all cancers, particularly cold tumours, highlighting the need for further research to reprogramme tumours to become more responsive to ICIs.

Martin-Ruiz et al. reviewed the effect of exercise (following inoculation with cancer cells) and nivolumab (anti-PD1) in NOD-SCID γ mice transplanted with human poorly-differentiated squamous non-small cell lung cancer (NSCLC), a hot tumour (37). A significantly raised necrotic index was observed in exercised mice with nivolumab compared with the non-exercise and isotype control group with the latter showing the greatest proliferating cell rate with Ki67 immunostaining (37). The necrotic index was not significantly different between the exercised and non-exercised groups receiving nivolumab and this may be due to the limited sample size (37). This study included combined aerobic and resistance training within the intervention, however we would be unable to draw any conclusions with regards to anti-cancer activity associated with resistance training alone. Proposed mechanisms of the anti-proliferative effects include promoting AMP-activated protein kinase (AMPK) signalling with subsequent suppression of the mammalian target of rapamycin (mTOR) oncogenic pathway (37, 43). A statistically significant reduction in key components of mTOR activation was observed in a study where human exercised serum was applied to A549 NSCLC cells (37, 43). Although no difference was seen in tumour growth or volume in mice receiving nivolumab irrespective of exercise, a reduced tumour growth rate and volume was found in the exercised mice in comparison to non-exercised mice, both groups having received isotype control (37). The immune deficient NOD-SCID γ mice would be unlikely to mount a sufficient response with anti-PD1 due to the inherent absence of T cells, providing difficulty in successfully evaluating the effect of exercise on cancer immunotherapy responses (44).

Buss et al. (44) examined EO771 ‘cold’ breast tumour and B16-F10 ‘hot’ melanoma mouse models and the interactions between post-implant exercise and anti-PD1 on local and systemic immunity, TME and growth. They observed a negative effect of exercise in these aggressive tumour models with a skew towards an immunosuppressive TME and exercise reduced CD8 T cell infiltration in the EO771 model and no effect on tumour growth seen when combined with anti-PD1 (44). Conversely, Wennerberg et al. demonstrated significant tumour growth inhibition with exercise during anti-PD-1 and radiotherapy treatment in a breast cancer murine model compared to sedentary mice receiving the same regimen (4, 45). They showed that exercise induced a local and systemic preference for anti-tumour immune cells and suppression of MDSC promoting the conversion of the breast cancer cold TME to a hot TME (4). Validity of the results and anti-PD1 used in Buss et al.’s study could be questioned as anti-PD1 alone exerted no tumour inhibitory effects and TME modulation also occurred with the isotype control (44). In clinical practice, pseudo-progression describes the inflammatory process with ICIs causing a paradoxical increase in assumed tumour volume, masking tumour response and may also provide a barrier to detecting the true anti-cancer effect of ICIs in murine models.

Further murine breast cancer models have also shown that utilising exercise alongside ICIs enhanced tumour suppression (41). In clinical practice, we utilise the combination of ICIs and chemotherapy for advanced triple negative breast cancer, however ICIs have shown little benefit outside of this sub-group, although multiple other treatments are available. Further studies into the role of ICI and exercise in breast cancer including hormone-positive and HER2-positive subgroups are required to support the clinical utility of exercise across all breast cancer sub-types.

Kurz et al. demonstrated that exercise optimised local immunity when combined with anti-PD1 by raising CD3 T cell and cytotoxic CD8+ T cell infiltrations and subsequently led to significant tumour size reduction compared to anti PD-1 alone in their PDA murine model (orthotopic transfer of KPC cells to wild type C57BL/6 mice) (27). The results are particularly promising due to the limited effective treatment for pancreatic cancer and the lack of responsiveness of cold tumours to immunotherapy, providing evidence that exercise could possibly expand the spectrum of tumours that could benefit from ICIs.

## 7 Discussion - incorporating exercise into clinical practice

### 7.1 Exercise is feasible in the advanced cancer population

This review has discussed the different mechanisms by which exercise training can reprogramme the cold TME with effector T cell infiltration, MDSC depletion, anti-tumour myokine secretion and tumour vasculature normalisation creating a hot TME.

Mouse models of both cold and hot tumours have showed additional benefits of combing immunotherapy with exercise training. Further exploration of this potentially advantageous combination is required with human studies. There is a clear unmet need in patients with advanced cancer to improve overall response to immunotherapy and survival outcomes. Within the advanced cancer population, reduced functional reserve, exercise tolerance and respiratory function may provide significant obstacles to implementing exercise training into their cancer management. It is unlikely there will be a generalised regime applicable to all patients with cancer. Therefore, there should be a role for exercise specialists, with expertise in working with patients with cancer, to be involved in the delivery of exercise interventions. Exercise protocols will need to be tailored to the individual patient’s needs and abilities, whilst ensuring intensity levels are reached to provide a sufficient immune response. This may not be possible in some cancer patients with frailty, cancer cachexia and disease burden potentially limiting their exercise abilities. Possible strategies to overcome these challenges could include utilising chair-based exercise activities, using a graded exercise intensity to build exercise tolerance and collaboration with nutritionists to minimise weight loss and maintain muscle mass.

However, the cancer population eligible for immunotherapy are inherently required to possess an Eastern Cooperative Oncology Group (ECOG) Performance Status of 0-1, indicating a good baseline level of activity and functioning (41). This further supports the feasibility of tailored exercise in these cohorts.

Mikkelsen et al. showed that strength training in combination with a home-based walking programme over 12 weeks was feasible in an elderly population affected by either advanced pancreatic cancer, biliary tract cancers or non-small cell lung cancer (46). They demonstrated a 69% adherence rate and 94% completion rate of attended sessions occurring alongside first line palliative oncological treatment (46). The acceptability of this exercise intervention by an older age population and the inclusion of hard-to-treat advanced cold tumours is promising for the future incorporation of exercise into clinical practice in these populations.

A combination of cycling and resistance training was incorporated into a rehabilitation programme for patients with advanced lung cancers including mesothelioma (47). Only one third of eligible patients managed to be recruited with a 56.6% completion rate of the 8-week programme (47). This highlights the need to review reasons behind reduced participant acceptability of exercise training, cater interventions, and acknowledge limitations within the advanced cancer population.

### 7.2 Moderate intensity aerobic exercise and resistance training promote anti-cancer activity

Importantly, to incorporate exercise as an evidence-based standard intervention in cancer management, the FITT principles allow prescription of a measured dose of exercise, although there will still undoubtedly be challenges in determining the exact ‘dose’ of exercise required to exhibit treatment effect. **Tables 1A**–**C** outline pre-clinical and clinical studies researching the relationship between exercise and cancer in cold and hot tumours. The *in vitro* studies required pre- and post- exercise conditioned serum of healthy participants or patients with cancer depending on the study (16, 22, 31, 32). The exercise regimes utilised varied from moderate –intensity to high-intensity interval training and largely centred on cycling. The window of opportunity studies (13, 14) reviewing exercise as a prehabilitation measure in oesophageal and pancreatic cancer patients, respectively, used a combination of aerobic exercise and resistance training at moderate intensity, with Bedoya et al. incorporating a walking activity over 2-6 months (14) and Zylstra et al. not specifying the type of aerobic exercise but occurring alongside neoadjuvant chemotherapy lasting 8-9 weeks (13).

**Table 1A:** Pre-clinical studies with cold tumours.

Study authors	Cancer type (s)	Cell line/animal model/human	Exercise intervention	Study findings
*In vitro*				
Orange et al. (16)	Colon	LoVo	Males with risk factors for colon cancer (n=16). Moderately intense cycling (5 minute sessions performed 6 times with 2.5 minute breaks)	Serum of exercised individuals significantly decreased LoVo cell proliferation and γ-H2AX expression compared to serum collected prior to exercise. Exercise led to increased IL-6 levels.
Aoi et al. (31)	Colon	C2C12 myocytes and colon-26	Healthy males 30 minutes uninterrupted cycling at 70% VO2 max Group 1: (n=10) One session Group 2: (n=9). Three weekly sessions for four weeks	Exercise increased SPARC levels in mice and humans following a single exercise session. SPARC reduced colon-26 cell proliferation.
Kim et al. (32)	Metastatic castrate-resistant prostate cancer (mCRPC)	DU145	mCRPC patients. Exercise group (n=13) 6 months of combination aerobic exercise (high-intensity, interval training) and resistance training. Control group (n=12) self-directed exercise	Supervised exercise training increased myokines oncostatin M (OSM) and SPARC compared to control. DU145 proliferation reduced with 6-month exercise-conditioned serum 12 to 61 hours after incubation but not at 72 hours.
Mouse studies				
Kurz et al. (27)	Pancreatic Ductal adenocarcinoma (PDA)	KPC cells in C57BL/6 mice	Running on a treadmill. 30 minutes, 5 times a week (mild intensity 15cm/s)	Exercise decreased tumour mass by 20-30%. Exercise increased intra-tumoural CD8+ T cells and reduced MDSCs with upregulation of 1L-15 signalling pathway.
Buss et al. (44)	Breast	Female C57BL/6 with orthotopic EO771 cells	Wheel running	Exercise did not affect NK cell levels. Exercise reduced CD8+ T cell proportions. Exercise combined with anti-PD1 had no significant effect on immune populations.
Wennerberg et al. (45)	Breast	4T1 in BALB/c mice	30 minutes 5 times a week. Running on a treadmill at 18m/min compared to control.	Exercise reduced tumour volume and MDSCs in the spleen. Exercise provided additional benefit in tumour inhibition when combined with radiotherapy and anti-PD1 compared to the latter two without exercise, as well as increased intratumoural NK cell activity.

**Table 1B:** Clinical studies with cold tumours.

Study authors	Cancer type (s)	Cell line/animal model/human	Exercise intervention	Study findings
Human studies				
Zylstra et al. (13)	Oesophageal cancer	Oesophageal cancer patients for possible curative surgery	Combined moderately intense aerobic exercise/weight training (n=21) compared to controls (n=19)	Enhanced pathological response and increased CD3 and CD8 T cells following pre-operative chemotherapy seen in the exercise group compared to control group.
Bedoya et al. (14)	PDA	Pancreatic cancer patients for possible curative surgery	70 patients undergoing neoadjuvant chemotherapy or combination chemotherapy/radiotherapy prior to surgery. Combination moderate level unsupervised aerobic exercise (1 hour per week of walking) and weight training (1 hour per week).	33/70 patients had their cancer surgery, although only 23 patients’ pathology specimens were able to be included. Compared to non-exercised controls, exercise increased microvessel density and patency in the tumours. Exercise did not influence the amount of tumour regression.

**Table 1C:** Hot tumours.

Study authors	Cancer type (s)	Cell line/animal model/human	Exercise intervention	Study findings
Mouse studies				
Pedersen et al. (12)	Melanoma, liver and lung	B16F10 in female mice, Diethylnitrosamine (DEN) in Naval Medical Research Institute NMRI male mice, Lewis Lung Carcinoma (LLC) in female mice	Wheel running. Average daily running distance per mouse: 4.1km (female) 6.8km (male)	All models: Tumour size, growth and metastatic disease reduced with exercise. B16 and LLC – increased pro- and anti-inflammatory immune components including IL-6 and NK cells.
Martin-Ruiz et al. (37)	Non-small cell lung cancer (NCSLC)	NOD-SCID gamma mice – patient derived xenograft model	40-60 minutes 5 times a week for 8 weeks. Combination treadmill running (performed at 40-80% maximal velocity) and resistance training.	Exercise led to reduced tumour growth and volume and in combination with nivolumab (anti-PD1), significantly increased the necrotic index comparative to double control, although necrotic index, cell proliferation and tumour growth were no different in the nivolumab groups irrespective of exercise.
Buss et al. (44)	Melanoma	Female C57BL/6 with subcutaneous B16F10 cells	Wheel running	Exercise did not affect NK cell levels. Exercise reduced CD8+ T cell proportions. Exercise combined with anti-PD1 had no significant effect on immune populations.

Animal studies in non-cancer models have shown that exercise training at moderate intensity, with a maximal oxygen uptake of around 70%, was able to promote cytotoxic immune function and support a Th1 cytokine profile, playing a protective role against infection (48). As immunity against infection overlaps with anti-tumour immunity, this further supports the use of moderate intensity exercise in cancer care.

Conversely, the opposite may be seen with chronic exercise at high intensity, for example in those at professional standards of exercise (48). After showing an initial increase within 30 minutes of high intensity endurance exercise, CD8+ T cells dropped along with Th1 cytokines IL2 and IFNγ (49). Previously, a 3-hour post exercise lymphopaenia had been addressed as potential redistribution from blood to tissue, however, a higher predisposition to developing infection has been noted (23, 49).

High intensity interval training was directly compared to moderate intensity continuous exercise over a 12-week programme in participants with significant risk factors for breast cancer in a randomised controlled trial (50). This study demonstrated a moderate intensity aerobic exercise regime over 12 weeks was superior to high intensity in increasing CD8 effector memory T cell proliferation and promoting improved immune responses by reducing senescence across different T cell subpopulations (50). Graff et al. were able to show that resistance training of the major muscle groups within a sedentary older age population exhibited a greater degree of leucocytosis, including cytotoxic CD8 T cells, than aerobic exercise (51). Pre-clinical and clinical evidence suggests the optimal exercise intervention to promote anti-cancer immunity would be moderate intensity, aerobic exercise training over a duration of 8-12 weeks with the inclusion of resistance training.

In the previously mentioned exercise studies, small sample sizes and significant heterogeneity in the study populations, exercise interventions and immune outcome measures limit our ability to draw firm conclusions. Furthermore, there may be selection bias of more physically active patients, who would be more likely to accept recruitment and contamination bias, due to the inability to prevent self-directed exercise in control groups. These would provide significant obstacles to defining the optimal exercise dose in patients with cancer.

Further clarification with larger studies focusing on a standardised effective exercise dose in a single disease site is required to allow implementation of exercise as a prescribed intervention in clinical practice.

Incorporating moderate intensity aerobic exercise for patients with cancer undergoing immunotherapy is likely to be more feasible and effective in promoting a favourable immune response. However, the translatability of these animal studies to patients with cancer should be considered given the differences in which an exercise intervention can be performed between humans and mice. Currently, there is limited evidence of the effectiveness of different exercise intensities within the advanced cancer population and is an area that warrants further exploration.

### 7.3 Future research

Despite the growing pre-clinical evidence of exercise-induced benefits on immunity and immunotherapy response in mouse models, there may be limitations in its translation to the human population. Notable differences in anatomy, physiology and immunology between mice and humans may limit extrapolating these beneficial effects of exercise interventions to humans. Differences in leucocyte subpopulations and Th1/Th2 profiles are present between mice strains as well as mice and humans (52). Therefore, importantly the utility of exercise as an effective intervention and an enhancer of immunotherapy responsiveness should be explored in ongoing human studies. ERICA is a prospective clinical trial looking into the practicalities and acceptability of undertaking exercise immediately prior to infusion of combination immunotherapy and chemotherapy in a French cohort of patients with metastatic NSCLC (53). Exploring the association between high intensity aerobic exercise and NK cell release in NSCLC, HI AIM is a randomised controlled trial that hypothesises augmented immunotherapy responses induced by exercise as a consequence of intratumoural and systemic immune cell influx (54). Importantly, these studies are investigating the clinical application of exercise in metastatic cancer populations, an area in oncology with vast room for improvement. NSCLC is typically a hot tumour and although there is scope for improving immunotherapy responses, to enhance the breadth of cancers that respond to immunotherapy, further research is required into cold tumours where immunotherapy is not effective.

Mechanisms by which drugs and oncolytic viruses can reprogramme cold tumours to become hot are being reviewed however, there is a research gap in demonstrating the ability of exercise to transform cold tumours to hot in patients with cancer. Future research could include ex-vivo work assessing immune responses when cold tumours are excised from patients and cultured with exercise-conditioned serum from participants undertaking moderate intensity aerobic exercise and resistance training.

**Figure 4:**
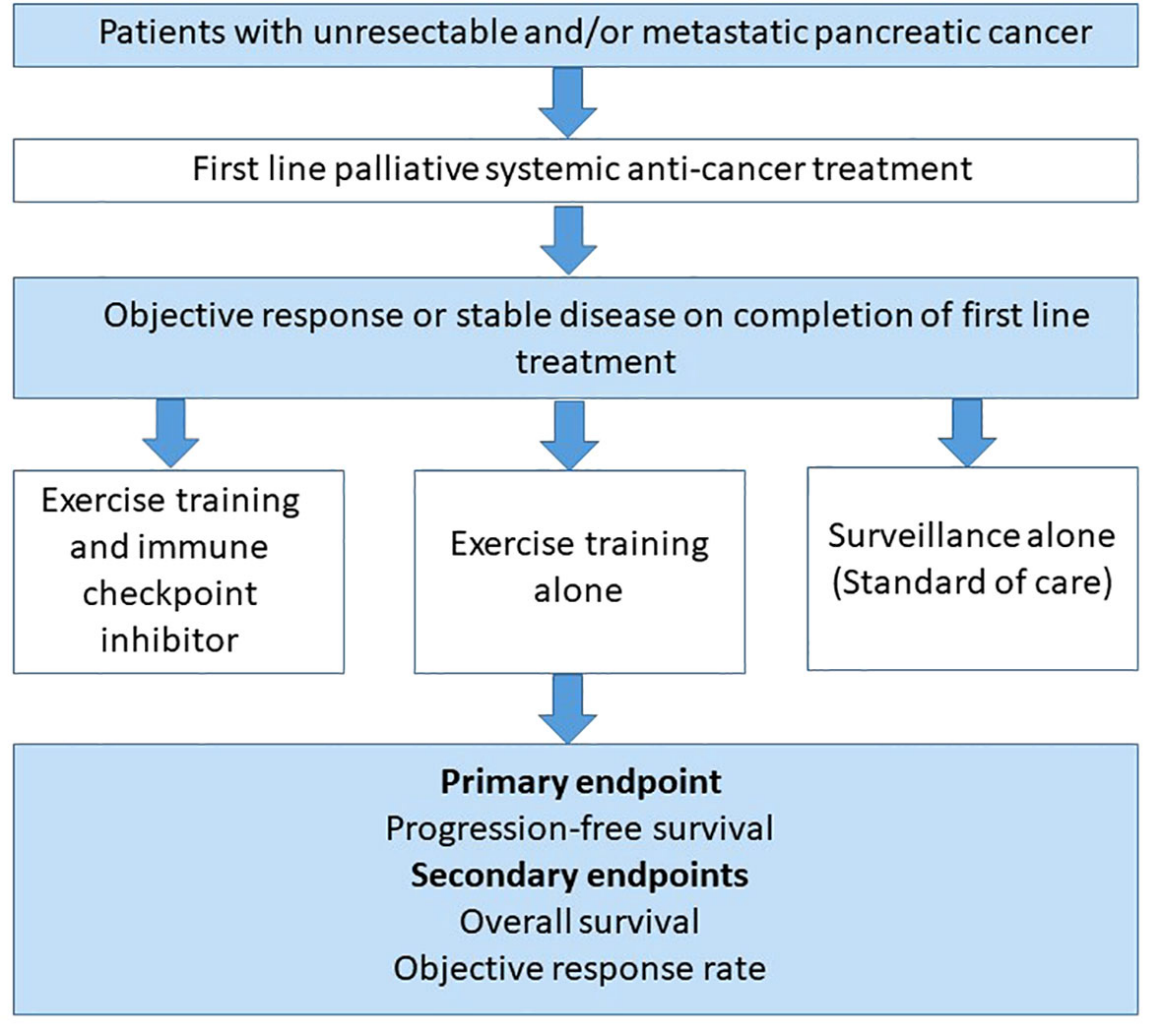
A multi-arm study design to explore the effectiveness of combining exercise training with immunotherapy as a maintenance strategy in previously treated advanced pancreatic cancer patients. A proposed randomised controlled trial design involving patients who have had an objective response or stable disease on completion of first line palliative systemic treatment for advanced pancreatic cancer. Patients would be randomised to either a combination of exercise training alongside a single agent immune checkpoint inhibitor or exercise training with standard of care or standard of care alone. All participants in each group will have regular Computed Tomography (CT) imaging every 3 months as standard of care. The primary endpoint would be progression-free survival (PFS), with overall survival (OS) and objective response rate (ORR) as secondary endpoints.

Due to lack of efficacy, immunotherapy alone cannot be used as a comparator to an exercise – immunotherapy combination in cold tumours. Therefore, a clinical study assessing the latter combination in malignancies such as advanced pancreatic cancer, could be assessed as a maintenance strategy in patients that have completed first line palliative systemic anti-cancer treatment and have either responded or have stable disease. If the patient remains well and deemed appropriate by the clinical team, they normally will undergo surveillance with regular Computed Tomography (CT) scans to assess for disease progression. **Figure 4** illustrates a potential multi-arm study design to explore exercise as an adjunct to immunotherapy in advanced pancreatic cancer in the maintenance setting following first line palliative treatment. Subsequently, if the combination of exercise and immunotherapy is deemed to be effective in the maintenance palliative setting, it can be explored at other stages and clinicians would be able to prescribe a dose of exercise alongside immunotherapy to improve treatment responses and overall survival.

## 8 Conclusion

There is an increasing volume of evidence supporting various exercise-induced direct cytotoxic mechanisms in different cancer settings along with a crucial role exercise may play in local tumour and systemic immunity via anti-tumour immune cell infiltrations, favourable cytokine and myokine profiles and tumour vasculature normalisation (9, 21, 30). In pre-clinical models, these different exercise-mediated changes appear to act synergistically to remodel the immunosuppressive cold TME to become hot.

Immunotherapy has improved survival outcomes for many patients with cancer although immunotherapy-resistance and severe immune-mediated toxicities limit its universal utility. A significant barrier to immunotherapy effectiveness lies within the rigid, immunosuppressive and hostile TMEs of cold tumours such as pancreatic cancer (5, 27). Evidence suggests that exercise training, including aerobic exercise and resistance training, may have a role in shifting tumours towards the hot end of the cold-hot TME spectrum. There is a need to validate these findings in humans.

Due to the complex relationship between different forms of exercise and immune components as well as the diverse nature of different cancers, defining an optimum dose of exercise to enhance immunity remains problematic, although studies suggest a favourable immune profile with moderate intensity aerobic exercise and resistance training. In practice, it is likely that exercise activities would need to be tailored to the individual, taking into account their personal limitations and their cancer phenotype. Large, clinical randomised controlled trials are needed to assess the utility of specific exercise interventions as adjuncts to immunotherapy across different cancer settings. Following this, if implemented into clinical practice, exercise training could potentially be a promising, safe and cost-effective measure to overcome some of the obstacles to optimising immunotherapy responsiveness.

## Author contributions

BH: Writing – original draft, Writing – review & editing. SD: Writing – review & editing. JW: Writing – review & editing. MM: Writing – review & editing.
